# Effects of a large-scale distribution of water filters and natural draft rocket-style cookstoves on diarrhea and acute respiratory infection: A cluster-randomized controlled trial in Western Province, Rwanda

**DOI:** 10.1371/journal.pmed.1002812

**Published:** 2019-06-03

**Authors:** Miles A. Kirby, Corey L. Nagel, Ghislaine Rosa, Laura D. Zambrano, Sanctus Musafiri, Jean de Dieu Ngirabega, Evan A. Thomas, Thomas Clasen

**Affiliations:** 1 London School of Hygiene and Tropical Medicine, London, United Kingdom; 2 Emory University Rollins School of Public Health, Emory University, Atlanta, Georgia, United States of America; 3 University of Arkansas for Medical Sciences, Little Rock, Arkansas, United States of America; 4 University of Rwanda School of Medicine and Pharmacy, Butare, Rwanda; 5 Rwanda Biomedical Center, Kigali, Rwanda; 6 East African Health Research Commission, Arusha, United Republic of Tanzania; 7 Mortenson Center in Global Engineering, University of Colorado Boulder, Boulder, Colorado, United States of America; Makerere University Medical School, UGANDA

## Abstract

**Background:**

Unsafe drinking water and household air pollution (HAP) are major causes of morbidity and mortality among children under 5 in low and middle-income countries. Household water filters and higher-efficiency biomass-burning cookstoves have been widely promoted to improve water quality and reduce fuel use, but there is limited evidence of their health effects when delivered programmatically at scale.

**Methods and findings:**

In a large-scale program in Western Province, Rwanda, water filters and portable biomass-burning natural draft rocket-style cookstoves were distributed between September and December 2014 and promoted to over 101,000 households in the poorest economic quartile in 72 (of 96) randomly selected sectors in Western Province. To assess the effects of the intervention, between August and December, 2014, we enrolled 1,582 households that included a child under 4 years from 174 randomly selected village-sized clusters, half from intervention sectors and half from nonintervention sectors. At baseline, 76% of households relied primarily on an improved source for drinking water (piped, borehole, protected spring/well, or rainwater) and over 99% cooked primarily on traditional biomass-burning stoves. We conducted follow-up at 3 time-points between February 2015 and March 2016 to assess reported diarrhea and acute respiratory infections (ARIs) among children <5 years in the preceding 7 days (primary outcomes) and patterns of intervention use, drinking water quality, and air quality. The intervention reduced the prevalence of reported child diarrhea by 29% (prevalence ratio [PR] 0.71, 95% confidence interval [CI] 0.59–0.87, *p* = 0.001) and reported child ARI by 25% (PR 0.75, 95% CI 0.60–0.93, *p* = 0.009). Overall, more than 62% of households were observed to have water in their filters at follow-up, while 65% reported using the intervention stove every day, and 55% reported using it primarily outdoors. Use of both the intervention filter and intervention stove decreased throughout follow-up, while reported traditional stove use increased. The intervention reduced the prevalence of households with detectable fecal contamination in drinking water samples by 38% (PR 0.62, 95% CI 0.57–0.68, *p* < 0.0001) but had no significant impact on 48-hour personal exposure to log-transformed fine particulate matter (PM_2.5_) concentrations among cooks (β = −0.089, *p* = 0.486) or children (β = −0.228, *p* = 0.127). The main limitations of this trial include the unblinded nature of the intervention, limited PM_2.5_ exposure measurement, and a reliance on reported intervention use and reported health outcomes.

**Conclusions:**

Our findings indicate that the intervention improved household drinking water quality and reduced caregiver-reported diarrhea among children <5 years. It also reduced caregiver-reported ARI despite no evidence of improved air quality. Further research is necessary to ascertain longer-term intervention use and benefits and to explore the potential synergistic effects between diarrhea and ARI.

**Trial registration:**

Clinical Trials.gov NCT02239250.

## Abstract 2

Thomas Clasen and colleagues report their trial in which reduced levels of diarrhea in children under 5 result from the introduction of water filters and rocket-style cookstoves in Rwanda.

## Author summary

**Why was this study done?:**

- Unsafe drinking water and household air pollution are leading health risks for young children in low-income countries and contribute to diarrhea and acute respiratory infections (ARIs), both of which are leading causes of child mortality and major burdens on healthcare systems.
- Systematic reviews have shown household-based water treatment to be effective in improving water quality and preventing diarrhea in low-income countries, while cooking indoors with solid fuels such as wood is associated with ARI in children.
- Intervention studies of household-based water treatment and higher-efficiency cookstoves have largely been short-term in duration, and there is limited evidence on intervention use, exposure, and health impacts when delivered programmatically at scale.

**What did the researchers do and find?:**

- In the context of a large-scale intervention program that delivered and promoted the use of an advanced water filter and portable biomass-burning “rocket” cookstove to over 101,000 low-income households in Rwanda, we conducted a cluster-randomized trial to assess intervention use, drinking water quality, and air quality (personal exposure to fine particulate matter, PM_2.5_) and diarrhea and ARI among children.
- A total of 1,582 households from 174 village-sized clusters were enrolled and visited approximately every 4 months for a year after receiving the intervention filter and stove.
- Among children under 5 years of age, the intervention reduced 7-day prevalence of diarrhea by 29% and ARI by 25%, although intervention use decreased throughout follow-up; drinking water quality improved, but adult and child personal exposures to PM_2.5_ remained unchanged and above WHO’s Air Quality Guideline (10 μg/m^3^) and Interim Target 1 (35 μg/m^3^) despite increased outdoor cooking.

**What do these findings mean?:**

- Our results suggest that this program was effective in reaching a vulnerable population, securing sustained use of the intervention, improving drinking water quality, and reducing risk of diarrhea and ARI among children under 5.
- While there was evidence of nonexclusive and diminished intervention use over time, our results suggest that the programmatic delivery and promotion of a combined household filter and cookstove intervention can provide a scalable interim solution among rural populations that currently lack access to safe piped-in drinking water, although cleaner cooking solutions are likely needed to optimize health benefits.
- Additional research is necessary to assess sustained benefits and identify effects, if any, on more serious cases of infection, as well as to explore the potential synergistic effects of combined household-level interventions.

## Introduction

Unsafe drinking water and household air pollution (HAP) are major causes of morbidity and mortality worldwide, particularly among young children in low-income countries [1]. Fecal contamination of drinking water contributes to diarrheal disease, the third leading cause of mortality in low-income countries, with an estimated 1.3 million deaths in 2015 [2]. An estimated 1.8 billion people worldwide lack access to safe drinking water, more than a third of whom rely primarily on water supplies such as shallow open wells and surface sources that are often highly contaminated with human and animal feces [3]. Cooking indoors with solid biomass fuels, such as wood, charcoal, and crop residues—the major source of HAP—is practiced by an estimated 3 billion people, primarily in low-income settings [4]. There is evidence linking the use of solid fuels to a wide range of health impacts, with the heaviest burden occurring among women and children, who spend the most time near cooking activities and experience the highest exposures [5].

In Rwanda, diarrhea and respiratory infection are the leading causes of childhood death after neonatal disorders and congenital anomalies, representing an estimated 10% and 15% of total child mortality, respectively [6]. The burden on the healthcare system is correspondingly heavy. According to the most recent Demographic Health Survey, the 2-week prevalence of diarrhea was 12% among children under 5, 44% of whom sought care at a health facility or healthcare provider [7]. In 2016, diarrhea and acute respiratory infections (ARIs) were the 2 main causes of morbidity among children under 5 years of age at health facilities, accounting for 16% and 24% of all cases, respectively [8]. Although ARIs are not generally as serious as acute lower respiratory infections (ALRIs), they pose a burden for children, their families, and healthcare providers [9–11].

Household water treatment offers the potential for reducing the risk of diarrhea and enteric infection. Boiling, filtering, chlorinating, or otherwise treating drinking water at the household level offers the potential of removing or killing fecal contaminants at the point of consumption. A systematic review of water quality interventions found that source-based interventions (other than piped water, for which there were no studies) were not protective against diarrhea; however, household filters reduced diarrhea by about half, with larger effects seen in trials of household filters with combined safe storage containers [12]. In general, however, the shorter-term (<6 month) trials reported larger effects, with longer trials showing little or no protective effects [12]. To date, no studies have examined the health impact of cookstove or water filter intervention programs implemented at scale in sub-Saharan Africa.

Improvements in household ventilation, fuel type, and stove type have been advocated as potential solutions to minimize the high exposures associated with solid fuel use and health impacts. However, there is mixed and limited evidence of the effect of “improved” (i.e., higher combustion efficiency) biomass-burning cookstoves on health [13]. Several recent trials examining the impact of more efficient, cleaner-burning cookstoves on child pneumonia or ALRIs have had mixed results [14,15]. A key challenge in these and other studies of cookstove interventions has been minimizing continued use of traditional stoves and obtaining, amidst multiple sources of exposure, the exposure reductions believed to be necessary for obtaining a health impact [16]. Relatively few studies to date have assessed the impact of stove programs on more broadly defined ARIs among children [17], even though these can be a burden on children and their families and can lead to or predispose others to more severe respiratory infections, including pneumonia [9,18,19].

The challenge of providing safe solutions for drinking water and cooking is especially great in sub-Saharan Africa, where most people continue to cook with solid fuel, and just 24% of the population has access to safely managed water supplies [20]. Although approximately 85% of Rwandans have access to improved water sources [20], this does not ensure that the water is safe for drinking [21]. A recent nationally representative assessment found that less than a quarter of household drinking water samples met World Health Organization (WHO) guidelines of having no detectable thermotolerant coliforms (TTCs), an indicator of fecal contamination [22]. According to the 2014–2015 Rwanda Demographic and Health Survey (DHS), an estimated 98% of households use solid biomass fuel for cooking, and most (78%) cook indoors [7].

We conducted this study to assess the health effects of a large-scale intervention of free provision and promotion of a portable biomass-burning natural draft “rocket” cookstove and advanced water filter in Western Province, Rwanda [23]. This paper reports on the impact of the intervention on reported diarrhea and ARIs in children <5 years of age, as well as household drinking water quality and personal exposure to fine particulate matter less than 2.5 micrometers in diameter (PM_2.5_) among cooks and children in a subset of households.

## Methods

### Intervention

The intervention program, which was branded “Tubeho Neza” (“Live Well” in Kinyarwanda), included free distribution of a tabletop gravity-based household water filter (the Vestergaard Frandsen LifeStraw Family 2.0) and a portable high-efficiency wood-burning cookstove (EcoZoom Dura), community and household education (e.g., via skits and radio songs), and behavior change messaging using culturally appropriate prepiloted materials informed by focus groups. The filter utilizes a gravity-fed ultrafiltration membrane, includes 5.5 liters of built-in safe storage with a tap, and has been classified by WHO as providing comprehensive protection against bacteria, viruses, and protozoa according to performance criteria [24]. Household members were encouraged to use the filter for all drinking water and the stove for all cooking activity. The program, which was financed by anticipated carbon credits [25], was implemented by the UK-based social enterprise DelAgua Health in collaboration with the Rwanda Ministry of Health (MOH) and targeted households in Ubudehe categories 1 and 2—roughly the government-designated poorest 25% of households in the province [26]. Previous pilot studies in similar settings found the filter and stove to be effective in improving drinking water quality and cooking area air quality [27,28].

Large-scale program activities in Western Province began on September 15, 2014. During the first year of the program, community health workers (CHWs) trained by the implementer (DelAgua Health and Rwanda MOH) delivered the intervention to all eligible households in 72 randomly allocated sectors, with the remaining 24 sectors assigned to serve as the control arm (no program activities). By the end of December, 2014, the program had reached over 101,000 households with an estimated 458,000 individuals. Repair (approximately 2.4% of all filters in first 12 months, 0.0% of stoves) and replacement (0.2% of all filters, 0.0% of stoves) was managed by DelAgua Health through district-level facilities and CHWs, who also undertook periodic filter and stove promotion activities through CHW cooperative meetings, community meetings, and household visits to intervention recipients and local officials participating in the program. Further details of the delivery of the interventions and associated program activities have been previously published [29].

### Study design

We assessed the health effects of the Tubeho Neza program using a cluster-randomized controlled trial design. Western Province is a largely rural province divided into 96 administrative sectors containing 3,612 villages, with a total population of about 2.5 million persons. Because the scale of the program precluded in-depth surveillance of each eligible household in the study area (*N* = 133,967), we selected a random sample of control and intervention households for baseline and repeated follow-up visits using two-stage cluster sampling. The trial design, setting, and characteristics of the study population have been previously described [23], and the protocol is available at https://www.sciencedirect.com/science/article/pii/S2451865416300266.

### Randomization

Allocation of sectors to receive the Tubeho Neza program was performed using a computer-generated randomization stratified by district to ensure equal distribution of the intervention across the 7 districts of Western Province [23]. Sectors were randomly assigned to the intervention or the control at a 3:1 ratio in order to meet implementer and MOH goals of delivery to 100,000 households. Randomization was performed by a member of the research team who was not involved in either data collection or intervention delivery, and sector assignments were provided to the implementers. Masking of participants or study enumerators was not possible because of the nature of the intervention. However, during study enrollment and baseline data collection, both study enumerators and participating households were blinded to intervention status.

### Sampling

Baseline and follow-up data were collected from a random sample of households in the study area. Households were eligible for inclusion if they met the implementer’s principal criterion for receipt of the intervention (household classified as Ubudehe category 1 or 2) and contained a child under the age of 4 years. We calculated the number of eligible households in each village in the study area using a list compiled by the implementer in collaboration with village officials prior to the start of the intervention delivery [23]. Villages that did not contain a sufficient number of eligible households to meet enrollment targets were merged with geographically contiguous villages using geographic information system software. We then conducted probability proportional to estimated size (PPES) random sampling of the resulting village clusters, with the estimated number of eligible households as the measure of cluster size [30]. We oversampled from the control sectors to yield a 1:1 ratio of intervention to control clusters, resulting in 87 intervention clusters containing 98 villages and 87 control clusters containing 101 villages. Within each of the 174 clusters, 10 eligible households were selected from the eligibility list using simple random sampling. Following household selection, trained enumerators located the selected households with the assistance of a village CHW. The primary point of contact for each household was the primary cook in that household. If the primary cook was not present at the first attempted visit of the household, another visit was attempted the same day, and a final attempt was made the following day. If the selected household was not present in the village or if contact was unsuccessful after the repeated attempts, an additional household in the village meeting the eligibility requirements was randomly selected for enrollment. Following baseline data collection, we constructed sampling weights to account for unequal selection probability due to household nonresponse, oversampling of control sectors, and variation in the number of children present in enrolled households [31].

### Baseline and follow-up visits

Prior to intervention delivery, we visited enrolled households to conduct a baseline survey and collect a drinking water sample. The smartphone-based survey included information about household demographic characteristics; reported cooking, heating, and lighting practices; stove observations; reported drinking water collection and treatment practices and observed hygiene and sanitation conditions based on core household survey questions from the WHO/UNICEF Joint Monitoring Programme for Water Supply and Sanitation [32]; reported consumption of unfiltered water in the previous 24 hours; and reported health. Following the baseline visit and delivery of the water filter and cookstove intervention, a total of 3 follow-up visits were conducted at approximately 4-month intervals by trained enumerators. Follow-up data collection consisted of household surveys, water quality testing, and air quality measurements.

### Primary and secondary health outcomes

The primary outcomes of this study were caregiver-reported diarrhea and ARI in the past 7 days among all children younger than 5 years, assessed using a structured survey tool. We used the WHO Integrated Management of Childhood Illness (IMCI) definition of diarrhea, which is 3 or more loose stools within a 24-hour period [33]. ARI was defined as illness with cough accompanied by rapid breathing or difficulty breathing [23]. A more restrictive ARI definition used by the Rwanda DHS 2014–2015 was examined as a secondary outcome and was defined as reported illness with cough in the previous 7 days accompanied by reported rapid breathing or difficulty breathing “due to a problem in the chest or nose and chest” [7].

In addition, we asked caregivers to report whether medical care for the reported episode of diarrhea or ARI was sought at a health facility or from a CHW, whether the child’s symptoms included visible blood in their stool, and whether the child had current or persistent diarrhea (defined as diarrhea with duration of at least 14 days). We also asked caregivers whether the child had received care for diarrhea or an ARI at a health clinic in the previous 2 months.

Current pneumonia, defined according to the IMCI protocol [34] and Rwanda’s Integrated Community Case Management (ICCM) protocol [35], was assessed as a tertiary outcome among children 2–59 months of age. Current IMCI pneumonia was defined as current illness with cough and difficulty breathing, accompanied by chest indrawing and/or rapid breathing ≥40 breaths/minute for children ≥12 months or ≥50 breaths/minute for children 2–12 months [34]. Respiratory rate was observed in a sleeping or calm child using the ARI 1-minute timer (UNICEF, Geneva, Switzerland). Current severe pneumonia or very severe illness among children 2–59 months of age was also defined according to IMCI and ICCM protocol as current illness with cough or difficulty breathing accompanied by severe symptoms (not able to drink, persistent vomiting, convulsions, lethargic/unconscious, stridor in a calm child, or severe malnutrition). Severe malnutrition was assessed by measuring middle-upper arm circumference (MUAC) for children ≥6 months and defined as a circumference <110 mm or the presence of edema in both feet for children 2–59 months [35]. Lastly, a reported burn from any stove within the previous 2 months was included as an additional outcome among children <5 years of age.

### Water quality

Drinking water quality was tested in each household at baseline and the first and second follow-up visits and analyzed as a secondary outcome. After administering the household survey, a 100 mL sample of the water a child under 5 would drink was collected, either directly from the water filter or from another storage container. If the child under 5 was reported to be too young to drink water, the water the respondent would drink was sampled. After collection, water samples were put on ice and processed within 8 hours after collection. Water samples were analyzed by study personnel at different locations throughout the province. TTC counts (colony-forming units per 100 mL) were obtained for each sample by trained laboratory staff using membrane filtration procedures prescribed for the Oxfam–DelAgua Incubator Kit (DelAgua, Wiltshire, UK).

### Air quality

Personal exposure to PM_2.5_ was measured among cooks and children using integrated gravimetric methods at baseline and all follow-up rounds, as described previously [23]. Measurements were conducted among 2 randomly selected households within 112 randomly selected clusters, half in the intervention group and half in the control group. To participate in the personal exposure sampling, the house had to be enrolled at baseline, have a primary cook who was a nonsmoker and was not currently pregnant, and include a child under 5 years of age who could support wearing measurement equipment weighing approximately 1 kg. The primary cook and a randomly selected child under 5 in each household meeting the eligibility criteria were instructed to wear the equipment for 48 hours or keep the equipment within 1 m when they were unable to wear it (e.g., during sleep, nursing, bathing, etc.). The gravimetric system consisted of a Casella TuffPro pump (Casella Measurement, Bedford, UK) set to 1.8 liters-per-minute flow at 1-minute intervals, connected by rubber tubing to a Harvard Personal Exposure Monitor (H-PEM) impactor (BGI, Cambridge, MA, USA), which was affixed within the breathing zone between chest and mouth and held by a diagonal strap for cooks and a small backpack for children. PM_2.5_ was collected on 37-mm diameter PTFE Teflo filters with 0.2-μm pore size and support ring (Pall Life Sciences, Port Washington, NY, USA), with Whatman drain disks for back support (Whatman GE Life Sciences, Pittsburgh, PA, USA). Flow was calibrated before and after sampling using a Challenger (BGI). The filters were weighed before and after deployment at Emory University (Atlanta, GA, USA). Filter field blanks (*n* = 14 filters) were collected throughout follow-up to account for cross-contamination during filter storage and handling, and the median (24 μg) was subtracted from all filter masses prior to concentration calculation.

### Coverage and use

Coverage and use of the intervention water filters and stoves were assessed as intermediate outcomes through a combination of self-report and direct observation by trained field enumerators. At each follow-up, the household primary cook was asked to identify and show the enumerator all stoves in the house and report on frequency and primary location of use. Enumerators then observed each stove’s type, current location, and whether it was currently being used, was currently warm, had marks of ash and looked recently used, or looked currently unused because of a dirty or dusty appearance and/or storage in an inconvenient location. Additionally, the primary cook was asked whether the intervention stove was in good working order (no reported or observed breakage resulting in nonuse), when it was last used, and the reasons for the stove being absent if the stove was not in the home at the time of the enumerator’s visit. The primary cook was also asked to identify the main drinking water container in the household and to report on usage and maintenance of the water filter. Additionally, the field staff documented whether the water filter was present in the home and assessed potential indicators of use, including whether the filter contained water at the time of the visit, whether the filter appeared to be in use (looked clean, was placed in a convenient place, or had a cloth on top of it), and whether the filter was in working order (no reported or observed breakage of filter parts resulting in nonuse).

### Statistical analyses

The sample size was calculated based on the primary outcomes of 7-day period prevalence of caregiver-reported diarrhea and ARI. We assumed a baseline diarrhea prevalence of 12% and a baseline ARI prevalence of 12% among children under 5 years in Western Province based on analysis of data from the 2010 Rwandan DHS and our pilot work in the study population [28]. We calculated the required sample size necessary to detect a 25% reduction in prevalence of each of diarrhea and ARI as being both clinically significant and consistent with previous studies examining the impact of household water treatment on diarrhea [12] and cookstoves on respiratory health. We assumed 10 children per village, 3 follow-up measures per child, a within-child (repeated measures) intracluster correlation (ICC) of 0.05, a within-village ICC of 0.02, and 15% follow-up loss, 80% power, and a significance level of α = 0.05 (two-sided test). A technical description of sample size calculations for this trial is reported elsewhere [23].

We analyzed child health outcomes using log-binomial regression with generalized estimating equations (GEEs) and robust standard errors to account for longitudinal sampling and village-level clustering. Models included a binary indicator of treatment allocation (control versus intervention) and were adjusted for age (in months) and gender. The model coefficients for treatment assignment were exponentiated to yield the prevalence ratios between the control and intervention arms [36]. Because counts of TTC in drinking water samples were both zero inflated and right-truncated, we categorized TTC into binary variables according to WHO risk category cutoff values (no detectable, ≤10 versus >10 TTC/100 mL, ≤100 versus >100 TTC/100 mL, and ≤1,000 versus >1,000 TTC/100 mL) and compared the prevalence of specified TTC thresholds between the control and intervention arms using log-binomial GEE models. Personal exposure to PM_2.5_ was natural-log transformed because of the skewed nature of the data and modeled using linear regression with GEEs and robust standard errors to account for clustering. Personal exposure models controlled for baseline exposure because of observed imbalance between arms at baseline (S1 Table). All models incorporated sampling weights to appropriately account for the two-stage PPES sampling design, baseline nonresponse, and oversampling of control sectors. Proportion of control and intervention household drinking water samples by level of fecal contamination TTC colony with 95% confidence intervals (CIs) were calculated using sampling weights and adjusted for village-level clustering. All statistical analyses were conducted using Stata 15 (Stata Corporation, College Station, TX, USA).

### Ethics

The study was reviewed and approved by the Ethics Committee of London School of Hygiene and Tropical Medicine (Ref #7711), the Institutional Review Board of Emory University (Ref #73615), the Rwanda National Ethics Committee Ref (#1497), and the National Health Research Committee of Rwanda (Ref #NHRC/2014/PROT/0163). Written informed consent was obtained from the primary cook (survey respondent) before baseline data collection. This trial is registered with ClinicalTrials.gov (NCT02239250).

## Results

### Enrollment and follow-up

**Fig 1:**
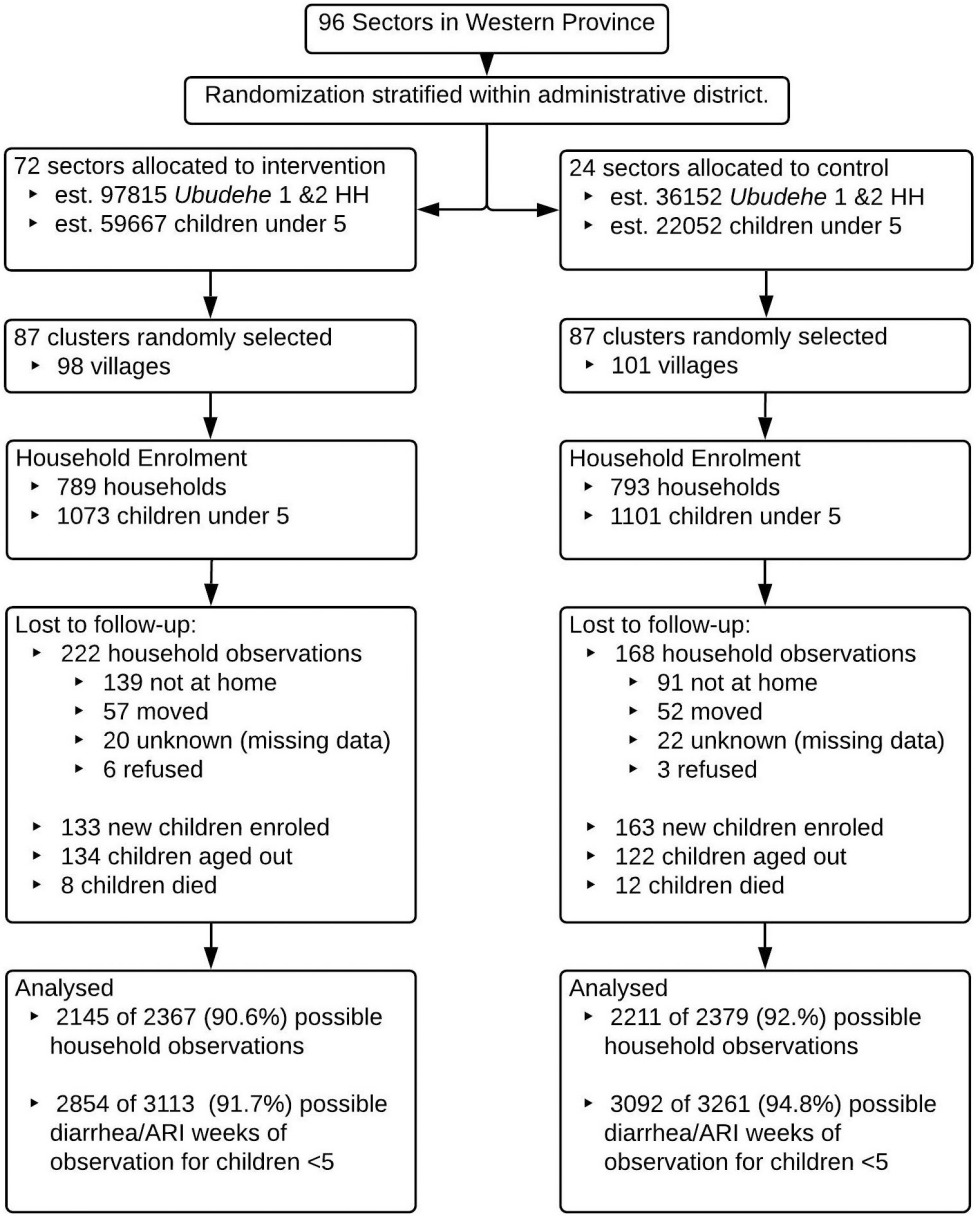
CONSORT flow diagram of enrollment and follow-up. ARI, acute respiratory infection; HH, household.

A total of 1,582 households were enrolled at baseline (August–December, 2014), 793 (50.1%) in the control arm and 789 in the intervention arm. The first follow-up visit occurred February–May, 2015 (Round 1), the second occurred June–September, 2015 (Round 2), and the third occurred October–March, 2016 (Round 3). All 174 clusters were followed up at each round. Data from 1,501 (94.9%) households were obtained at Round 1, 1,448 (91.5%) at Round 2, and 1,407 (88.9%) at Round 3. Follow-up and reasons for household attrition were similar between the intervention and control arms (Fig 1). A total of 2,440 children <5 years of age were enrolled into the study at baseline and throughout follow-up: 1,250 (51.2%) in the control arm and 1,190 (48.8%) in the intervention arm. Follow-up and reasons for attrition were similar between the intervention and control arms (Fig 1). Baseline household, respondent, and child characteristics were similar between the control and intervention arms (Table 1). Baseline diarrhea prevalence among children less than 5 years of age was 15.3% in the intervention arm and 13.7% in the control arm. Baseline ARI prevalence was 10.3% among children in the intervention arm and 10.5% among children in the control arm (Table 1).

**Table 1:** Respondent, household, and child characteristics at baseline.

	Control (%)	Intervention (%)
	*N* = 793 Households	*N* = 789 Households
**Respondent Characteristics**		
Mean age (SD)	32.9 (10.1)	32.7 (10.4)
Female	790/793 (99.6)	787/789 (99.7)
Attended school	579/793 (73.0)	582/789 (73.8)
**Household Characteristics**		
Mean # of household residents (SD)	5.3 (1.8)	5.2 (1.9)
Owns house	707/793 (89.2)	711/789 (90.1)
Has electricity	48/793 (6.1)	48/789 (6.1)
Owns mobile phone	311/793 (39.2)	338/789 (42.3)
Owns livestock	434/793 (54.7)	402/788 (51.0)
Floor material: earth/sand	757/793 (95.5)	757/789 (96.0)
Mean number of rooms (SD)	4.0 (1.3)	4.0 (1.3)
**Sanitation/Hygiene Facilities**		
Toilet facility: pit latrine without slab	416/712 (58.4)	454/716 (63.4)
Toilet facility located in plot	635/759 (83.7)	655/759 (86.3)
Shares toilet facility	206/765 (26.9)	205/762 (26.9)
Has handwashing location (specific place after defecation)	24/793 (3.0)	19/789 (2.4)
**Drinking Water Source/Practices**		
Primary water source		
Protected spring/well	393/793 (49.6)	452/789 (57.3)
Unprotected spring/well	156/793 (19.7)	134/789 (17.0)
Surface water	48/793 (6.1)	40/789 (5.1)
Primary water source: improved	589/793 (74.3)	615/789 (77.9)
Round trip time to water source <30 minutes	389/791 (49.2)	417/789 (52.9)
Reported use of household water treatment		
None	660/793 (83.2)	633/789 (80.2)
Boiling	119/793 (15.0)	106/789 (13.4)
Chlorination	11/793 (1.4)	22/789 (2.8)
Treat water every day	11/793 (1.4)	16/789 (2.0)
**Cooking, Lighting, and Heating**		
Current primary fuel: wood	333/793 (42.0)	459/789 (58.2)
Current primary fuel: straw/shrubs/grass	436/793 (55.0)	309/789 (39.2)
Current primary fuel: charcoal	18/793 (2.3)	17/789 (2.2)
Cooks 1 meal per day (%)	231/793 (29.1)	214/789 (27.1)
Cooks 2 meals per day (%)	507/793 (63.9)	500/789 (63.4)
Primary cooking location: inside house, kitchen room	321/792 (40.5)	276/789 (35.0)
Primary cooking location: outside	50/792 (6.3)	42/789 (5.3)
Primary cooking location: inside separate kitchen	280/792 (35.4)	322/789 (40.8)
Has traditional three-stone fire and uses everyday	601/754 (79.7)	618/765 (80.8)
Has built-in wood-burning stove (without chimney) and uses everyday	138/754 (18.3)	133/765 (17.4)
Uses kerosene for lighting every day	84/793 (10.6)	71/789 (9.0)
Heated home in last 7 days	121/793 (15.3)	94/789 (11.9)
**Household Drinking Water Quality**		
No detectable TTCs	161/681 (23.6)	175/651 (26.9)
1–10 TTC/100 mL	124/681 (18.2)	131/651 (20.1)
11–100 TTC/100 mL	139/681 (20.4)	118/651 (18.1)
>100 TTC/100 mL	257/681 (37.7)	227/651 (34.9)
**Child under 5 Years of Age Characteristics**	***N* = 1,101 Children**	***N* = 1,073 Children**
Female	548/1,101 (49.8)	544/1,073 (50.7)
Mean age (SD)	27.0 (15.5)	27.6 (15.4)
Completed pneumococcal vaccination (3-dose series observed on vaccination card)	754/1,101 (68.5)	683/1,073 (63.7)
Completed rotavirus vaccination (3-dose series observed on vaccination card)	489/1,101 (44.4)	405/1,073 (37.7)
Child or other household member has health insurance	615/1,101 (55.9)	599/1,073 (55.8)
Caretaker-reported 7-day diarrhea	150/1,098 (13.7)	164/1,073 (15.3)
Caretaker-reported 7-day ARI	114/1,090 (10.5)	109/1,058 (10.3)

**Abbreviations**: ARI, acute respiratory infection; SD, standard deviation; TTC, thermotolerant coliform.

### Intervention coverage

Within the intervention arm, the mean length of time between receipt of the intervention filter and stove and follow-up visit was 4.1 (SD 1.2, range 1.7–7.6) months at Round 1, 8.3 (SD 1.2, range 6.0–11.7) months at Round 2, and 13.4 (SD 1.7, range 10.1–17.5) months at Round 3. More than 96% of the intervention households reported receiving the filter and stove, with 94.5% of households reporting that they still had the filter and 95.2% reporting that they still had the stove in Round 3 (Table 2). The filter and stove were reported to be functioning in 91.8% and 95.5% of household visits, respectively. In Round 3, the most common reasons for not having the filter (if they had received it) were giving, lending, or selling the filter (9 households), theft (5 households), and breakage (3 households), while the most common reasons for not currently having the stove were giving or lending the stove to a neighbor (6 households), theft (5 households), selling it (1 household), or storing it in another location (1 household).

**Table 2:** Intervention filter and stove coverage and observed and reported use by follow-up round and overall.

	Round 1	Round 2	Round 3	Overall
	*N* (%)	*N* (%)	*N* (%)	(Round 1–3)
	*N* = 739 hh	*N* = 718 hh	*N* = 688 hh	*N* = 2,145 hh Observations
**Coverage: Intervention Filter and Stove**				
Received filter: reported	715 (96.8)	695 (96.8)	668 (97.1)	2,078 (96.9)
Currently has filter: reported	707 (95.7)	683 (95.1)	650 (94.5)	2,040 (95.1)
Currently has functioning filter: reported^a^	697 (94.3)	665 (92.6)	607 (88.2)	1,969 (91.8)
Received intervention stove: reported	715 (96.8)	695 (96.8)	668 (97.1)	2,078 (96.9)
Currently has stove: reported	708 (95.8)	687 (95.7)	655 (95.2)	2,050 (95.6)
Currently has functioning stove: reported^b^	708 (95.8)	687 (95.7)	653 (94.9)	2,048 (95.5)
**Observed and Reported Use: Intervention Filter**				
Filter observed at the household	695 (94.0)	672 (93.6)	632 (91.9)	1,999 (93.2)
Filter observed and reports last filled since yesterday	465 (62.9)	430 (59.9)	329 (47.8)	1,224 (57.1)
Filter observed and reports last filled since day before yesterday	560 (75.8)	508 (70.8)	432 (62.8)	1,500 (69.9)
Filter observed and reports last filled within previous week	625 (84.6)	583 (81.2)	489 (71.1)	1,697 (79.1)
Observed filter looks in use	558 (75.5)	562 (78.3)	446 (64.8)	1,566 (73.0)
Observed filter contains water	489 (66.2)	458 (63.8)	374 (54.4)	1,321 (61.6)
**Observed and Reported Treatment of hh Drinking Water Sample**				
Currently has drinking water in hh	655 (88.6)	663 (92.3)	536 (77.9)	1,854 (86.4)
Drinking water reportedly treated by filter	456 (61.7)	431 (60.0)	371 (53.9)	1,258 (58.6)
Drinking water stored in filter	375 (50.7)	391 (54.5)	343 (49.9)	1,109 (51.7)
**Observed and Reported Use: int. and trad Stoves**				
Able to observe hh’s stoves	736 (99.6)	713 (99.3)	680 (98.8)	2,129 (99.3)
Int. stove observed at the hh	702 (95.0)	678 (94.4)	639 (92.9)	2,019 (94.1)
Int. stove has appearance of use	656 (88.7)	620 (86.4)	488 (70.9)	1,764 (82.2)
Trad stove has appearance of use	494 (66.8)	526 (73.3)	520 (75.6)	1,540 (71.8)
Int. stove observed and reports last used since yesterday	521 (70.5)	489 (68.1)	374 (54.4)	1,384 (64.5)
Int. stove observed and reports last used since day before yesterday	600 (81.2)	563 (78.4)	443 (64.4)	1,606 (74.9)
Reported int. stove use: twice a week or more	677 (91.6)	635 (88.4)	522 (75.9)	1,834 (85.5)
Reported trad stove use: twice a week or more	388 (52.5)	448 (62.4)	474 (68.9)	1,310 (61.1)
Reported int. stove use: every day	539 (72.9)	503 (70.1)	361 (52.5)	1,403 (65.4)
Reported trad stove use: every day	178 (24.1)	237 (33.0)	340 (49.4)	755 (35.2)
Int. stove in use or warm at visit	109 (14.7)	120 (16.7)	85 (12.4)	314 (14.6)
Trad stove in use or warm at visit	176 (23.8)	136 (18.9)	187 (27.2)	499 (23.3)
Int. stove in use at visit	77 (10.4)	71 (9.9)	48 (7.0)	196 (9.1)
Trad stove in use at visit	126 (17.1)	91 (12.7)	123 (17.9)	340 (15.9)
Int. stove in use outdoors at visit	28 (3.8)	39 (5.4)	25 (3.6)	92 (4.3)
Int. stove reportedly used primarily outdoors	383 (51.8)	449 (62.5)	350 (50.9)	1,182 (55.1)
Primary cooking location outdoors (all stoves)	298 (40.3)	313 (43.7)	196 (28.5)	807 (37.6)

^a^hh received LifeStraw 2.0 intervention filter and reports it currently present at the hh, with no reported/observed breakage resulting in nonuse.

^b^hh received EcoZoom Dura intervention stove and reports currently present at the hh, with no reported/observed breakage resulting in nonuse.

**Abbreviations**: hh, household; int., intervention; trad, traditional.

### Reported and observed filter use

Over 75% of households in the intervention group had filters meeting the study criteria for apparent use in Round 1, which decreased to 64.8% in Round 3; water was observed in 66.2% (489/739) and 54.4% (374/688) of filters in Rounds 1 and 3, respectively. Similarly, drinking water observed by enumerators was reportedly treated with the filter in 61.7% (456/739) of households in Round 1 and 53.9% (371/688) in Round 3. In addition to declines in filter functionality and use, among intervention respondents who reported that they were a water drinker and currently had the filter, 23.3% (161/691) reported drinking unfiltered water within the previous 24 hours in Round 1, increasing to 34.4% (211/614) in Round 3. In households with the filter and a child under 5 reported to be a water drinker in Round 1, 20.8% (141/679) of households reported that the child had consumed unfiltered water within the previous 24 hours, increasing slightly to 25.1% (146/581) in Round 3. In the same households, 3.1% (21/679) had a child who was reported to be consuming unfiltered water every day in Round 1, and this increased to 11.5% (67/581) in Round 3.

### Reported and observed stove use

At Round 1, 81.2% (600/739) households reported last using the intervention stove within the previous 2 days, which declined to 64.4% by Round 3 (Table 2). Frequency of reported use also declined from Round 1 to Round 3, with everyday use declining from 72.9% to 52.5%, while everyday use of a traditional stove rose from 24.1% to 49.4%. Overall, reported use of the intervention stove twice per week or more (85.5%) was higher than everyday use (65.4%), as was traditional stove use (61.1% used twice per week or more versus 35.2% used every day). While reported use of the intervention stove declined over time, reported use primarily outdoors was fairly consistent across rounds, with 51.8% reporting the practice in Round 1 and 50.9% in Round 3 (Table 2); overall, 37.6% of intervention households reported their primary cooking area was outdoors, compared to 5.3% at baseline.

Overall, we were able to observe household stoves at 99.3% (2,129/2,145) of households (Table 2). In Round 1, 88.7% of households met the study criteria for apparent use of the intervention stove, and this decreased to 70.9% in Round 3. However, 66.8% of households had a traditional stove that met study criteria for apparent use in Round 1, increasing to 75.6% in Round 3; 17.1% of households had a traditional stove that was currently in use (lit) in Round 1 and 17.9% at Round 3 (Table 2).

### Water quality

**Fig 2:**
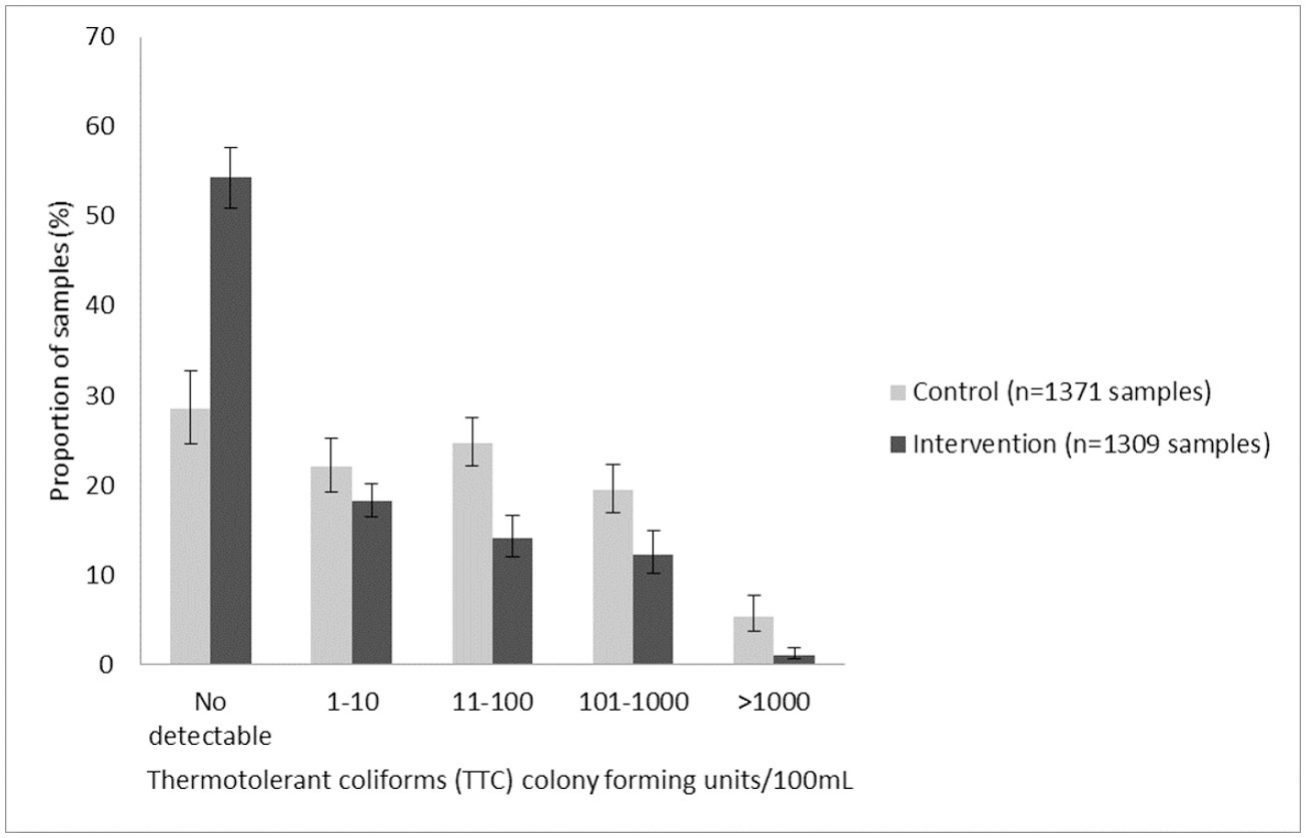
Proportion of control and intervention household drinking water samples by level of fecal contamination (TTC colony-forming units per 100 mL), with 95% CIs, adjusted for clustering and sampling design. CI, confidence interval; TTC, thermotolerant coliform.

A total of 2,680 samples of drinking water were collected and analyzed: 1,371 (51.2%) from control households and 1,309 from intervention households (Fig 2). In the intervention group compared to the control group, there was a 38% reduction in the prevalence of detectable TTC contamination present in drinking water samples (prevalence ratio [PR] 0.62, 95% CI 0.57–0.68, *p* < 0.0001). There was also a 46% reduction in the prevalence of moderate and higher contamination (>10 TTC/100 mL) among samples from the intervention group as compared to samples from the control group (PR 0.54, 95% CI 0.46–0.63, *p* < 0.0001), a 50% reduction in the prevalence of high contamination (>100 TTC/100 mL) (PR 0.50, 95% CI 0.39–0.65, *p* < 0.0001), and an 81% reduction in the prevalence of very high contamination (>1,000 TTC/100 mL) (PR 0.19, 95% CI 0.10–0.38, *p* < 0.0001). In the intervention arm, 521/762 (68.4%) drinking water samples stored in the filter were free of TTCs, while 190/547 (34.7%) stored in another storage container were free of TTC contamination.

### Air quality

After excluding unreliable records due to incomplete sampling periods (<90% of 48 hours), pump flow, missing weights, and noncompliance, a total of 231 measures of primary cook exposure and 159 measures of child exposure were usable in follow-up analyses. Mean 48-hour time-integrated PM_2.5_ exposure among the cooks was 218 μg/m^3^ (median 158 μg/m^3^, IQR 97–251 μg/m^3^) in the intervention arm and 223 μg/m^3^ (median 146 μg/m^3^, IQR 94–298 μg/m^3^) in the control arm. Natural log PM_2.5_ was not significantly different between arms (β = −0.089, *p* = 0.486, *n* = 183 model observations), controlling for baseline PM_2.5_. Among the children, mean PM_2.5_ exposure was 224 μg/m^3^ (median 154 μg/m^3^, IQR 85–267 μg/m^3^) in the intervention arm and 231 μg/m^3^ (median 161 μg/m^3^, IQR 91–285 μg/m^3^) in the control arm. Similarly, there was no significant difference in natural log PM_2.5_ between groups (β = −0.228, *p* = 0.127, *n* = 84 model observations) controlling for baseline PM_2.5_.

### Health outcomes

Overall, the prevalence of caregiver-reported diarrhea in the previous 7 days among children under 5 years of age during follow-up was 8.7% (249/2,849) in the intervention arm and 12.9% (397/3,086) in the control arm, representing a 29% reduction in prevalence after adjusting for age and gender (PR 0.71, 95% CI 0.59–0.87, *p* = 0.001) (Table 3). The within-village ICC was 0.02, and the within-child ICC was 0.09. Among diarrhea-related secondary outcomes, there was a nonsignificant reduction in the reported 1-week prevalence of seeking care for diarrhea from a CHW (PR 0.68, 95% CI 0.44–1.06, *p* = 0.089), and a significant reduction in reported 1-week prevalence of seeking care from a health facility (PR 0.54, 95% CI 0.32–0.91, *p* = 0.020) (Table 3). The prevalence of a reported visit to a health facility for diarrhea within the previous 2 months was also lower in the intervention arm (PR 0.72, 95% CI 0.54–0.96, *p* = 0.023). Although there was lower prevalence of reported bloody dysentery, persistent diarrhea, and current diarrhea in the intervention arm, overall prevalence was very low for these secondary outcomes (≤2.7%), and there were no statistically significant differences at the *p* = 0.05 level (*p* = 0.369, *p* = 0.119, and *p* = 0.411, respectively) (Table 3).

**Table 3:** Health outcomes during follow-up for children younger than 5 years of age with prevalence ratios adjusted for age in months and gender.

	Intervention	Control	PR^a^ (95% CI)	*p*
	*n* (%)	*n* (%)		
	*N* = 2,854 Child Observations	*N* = 3,092 Child Observations		
**Primary Outcome**				
7-day diarrhea	249/2,849 (8.7)	397/3,086 (12.9)	0.71 (0.59, 0.87)	0.001
7-day ARI^b^	283/2,850 (9.9)	441/3,084 (14.3)	0.75 (0.60, 0.93)	0.009
**Secondary Outcomes: Diarrhea**				
7-day CHW visit for diarrhea	51/2,835 (1.8)	93/3,068 (3.0)	0.68 (0.44, 1.06)	0.089
7-day health facility visit for diarrhea	27/2,835 (1.0)	53/3,068 (1.7)	0.54 (0.32, 0.91)	0.020
7-day bloody dysentery	30/2,851 (1.1)	46/3,084 (1.5)	0.77 (0.43, 1.37)	0.369
7-day persistent diarrhea^c^	1/2,601 (0.0)	7/2,696 (0.3)	0.19 (0.02, 1.54)	0.119
Current diarrhea (IMCI)	67/2,853 (2.3)	83/3,092 (2.7)	0.86 (0.60, 1.23)	0.411
**Secondary Outcomes: Respiratory**				
7-day CHW visit for ARI	60/2,807 (2.1)	111/3,029 (3.7)	0.62 (0.40, 0.98)	0.039
7-day health facility visit for ARI	51/2,807 (1.8)	54/3,029 (1.8)	1.07 (0.68, 1.69)	0.760
7-day ARI: DHS definition^d^	103/2,795 (3.7)	161/2,989 (5.4)	0.75 (0.51, 1.10)	0.140
7-day CHW visit for ARI (DHS)	32/2,796 (1.1)	60/2,992 (2.0)	0.65 (0.36, 1.17)	0.151
7-day health facility visit for ARI (DHS)	35/2,796 (1.3)	29/2,993 (1.0)	1.33 (0.75, 2.34)	0.329
Current pneumonia (IMCI)^e^	41/2,574 (1.6)	55/2,829 (1.9)	0.87 (0.58, 1.30)	0.491
Current severe pneumonia or very severe illness (IMCI)^f^	26/2,574 (1.0)	40/2,829 (1.4)	0.75 (0.45, 1.24)	0.256
**Other Outcomes**				
Visit to health facility for diarrhea within the last 2 months	154/2,851 (5.4)	217/3,091 (7.0)	0.72 (0.54, 0.96)	0.023
Visit to health facility for ARI within the last 2 months	282/2,851 (9.9)	304/3,089 (9.8)	0.99 (0.79, 1.25)	0.950
Burn within the last 2 months	51/2,850 (1.8)	112/3,090 (3.6)	0.51 (0.36, 0.74)	<0.001

^a^PR, 95% CIs, and *p*-values derived from weighted log-binomial GEE models adjusted for age and gender.

^b^ARI defined as reported cough accompanied by reported rapid breathing or difficulty breathing

^c^ Persistent diarrhea defined as diarrhea lasting 14 or more days.

^d^ARI: DHS definition defined as reported illness with cough accompanied by reported rapid or difficulty breathing due to problem in the chest or nose and chest.

^e^Current pneumonia (IMCI) defined as current illness with cough and difficulty breathing, accompanied by rapid breathing ≥40 breaths/min for children ≥12 months, or ≥50 breaths/min for children 2–12 months. Does not include children <2 months.

^f^Current severe pneumonia or very severe illness (IMCI) defined as current illness with cough or difficulty breathing accompanied by severe symptoms (not able to drink, persistent vomiting, convulsions, lethargic/unconscious, stridor in a calm child, or severe malnutrition). Does not include children <2 months.

**Abbreviations**: ARI, acute respiratory infection; CHW, community health worker; CI, confidence interval; DHS, Demographic and Health Survey; GEE, generalized estimating equation; IMCI, Integrated Management of Childhood Illness; PR, prevalance ratio.

The prevalence of caretaker-reported ARI in the previous 7 days among children less than 5 years of age during follow-up was 9.9% in the intervention arm and 14.3% in the control arm, representing a 25% reduction in prevalence after adjusting for age and gender (PR 0.75, 95% CI 0.60–0.93, *p* = 0.009) (Table 3). The within-village ICC was 0.04, and the within-child ICC was 0.12. Among respiratory-related secondary outcomes, there was a 38% reduction in reported seeking of care from a CHW for ARI (PR 0.62, 95% CI 0.40–0.98, *p* = 0.039) but no significant difference for seeking of care at a health facility for ARI in the previous week (PR 1.07, *p* = 0.760) or previous 2 months (PR 0.99, *p* = 0.950). There were fewer cases of reported DHS-defined ARI in the past week than the broader ARI case definition in both groups, and the reduction in cases we observed among the intervention group was not statistically significant (PR 0.75, 95% CI 0.51–1.10, *p* = 0.140). Similarly, we observed no significant intervention-related effects on seeking care from a CHW for DHS-defined ARI (PR 0.65, 95% CI 0.36–1.17, *p* = 0.151) or seeking care at a health facility in the previous week (PR 1.33, 95% CI 0.75–2.34, *p* = 0.329) (Table 3).

Current pneumonia, according to WHO IMCI criteria, was diagnosed by the enumerators in observations of 41/2,574 children in the intervention group (1.6%) and in 55/2,829 children in the control group (1.9%). After adjusting for age and gender, this difference was nonsignificant (PR 0.87, 95% CI 0.58–1.30, *p* = 0.491). There was also no statistically significant reduction of current WHO-defined severe pneumonia/very severe illness between groups (PR 0.75, 95% CI 0.45–1.24, *p* = 0.256). Prevalence of reported burns within the previous 2 months was lower in the intervention arm compared to controls, with 1.8% prevalence in the intervention arm and 3.6% prevalence in the control arm (PR 0.51, 95% CI 0.36–0.74, *p* < 0.001).

## Discussion

We assessed the impact of a large-scale program in Rwanda that provided and promoted the use of a tabletop gravity-based water filter and portable natural draft “rocket stove” to households in the poorest socioeconomic quartile. Ten to 17 months after delivery of the intervention, most households had the filter and reported using it, and approximately half of households possessed filters that were observed to contain water. However, we recorded evidence of decreasing use over time, and consumption of untreated water was reported among some respondents and children. The intervention was effective in improving household drinking water quality and reducing the risk of reported diarrhea in children <5 years old by 29% (95% CI 13%–41%). Notably, the intervention reduced reported diarrhea-related child visits to health facilities within the previous week, a possible indication of effects on severe episodes [37]. A majority of households reported using the intervention stove 10 to 17 months after delivery, although reported use decreased over time, and traditional stove use persisted and increased over time. The intervention reduced the risk of reported ARI by 25% (95% CI, 7%–40%), as well as reported visits to CHWs by 38% despite the lack of an effect on the household primary cook’s or child’s exposure to PM_2.5_.

The levels of filter coverage and use achieved in the intervention group were generally higher than those reported by other programmatically-delivered household water treatment interventions. Evaluations of larger studies of solar disinfection (SODIS) and chlorine for disinfecting water achieved low levels of uptake [38,39]. An assessment of a large-scale program in Kenya of the first-generation hanging version of the LifeStraw filter also showed poor and declining coverage and use [40]. It is possible that filters, which have the potential of improving drinking water aesthetics, are more acceptable to households than other forms of household water treatment and that the tabletop configuration of the filter used in this case was more acceptable to householders than previous versions. The intervention’s impact may also be due in part to extensive planning and piloting; the involvement of CHWs; engagement with local, regional, and national leaders; the development of culturally appropriate training and promotional materials, including skits and radio songs; and incorporating feedback from community focus groups into programmatic activities [29]. A previous study of the pilot intervention in Rwanda suggests filter use can persist 1–2 years after receipt in the context of ongoing programmatic engagement [28].

The impact of the intervention on diarrhea is generally consistent with that of pooled estimates from other studies of household-based filtration of drinking water. Three previous trials of the first-generation LifeStraw filter yielded a pooled relative risk of 0.69 (95% CI, 0.51–0.93; 3,259 participants, low-quality evidence) [12], similar to that observed in this study. On the other hand, the diarrhea results are in contrast with those from 3 rigorous efficacy trials of point-of-use chlorination interventions in Bangladesh, Kenya, and Zimbabwe that showed no effect on diarrhea in children <5 [41–43]. One possible reason for the difference may be that unlike the LifeStraw filter, which is effective against bacteria, viruses, and parasites, those 3 trials employed chlorine-based disinfectants that are not effective against *Cryptosporidium* spp., which recent evidence has shown to be a leading cause of moderate to severe diarrhea in Bangladesh and Kenya [44]. A recent study found that among children who were age-eligible to receive the rotavirus vaccine, *Cryptosporidium* spp. had the second-highest attributable fraction for acute diarrhea in sub-Saharan Africa [45], and globally, it is estimated to be the second-highest cause of diarrhea-related mortality among children under 5 [2]. Lower seroprevalence of immunoglobulin G (IgG) antibody response to *Cryptosporidium parvum* at follow-up in intervention children under 2 years of age enrolled in our trial (relative risk 0.62, 95% CI 0.44–0.89) [46] also suggests the impact of the filter on diarrhea may be due in part to its effectiveness against this pathogen.

The reported respiratory-related health impacts are surprising given the continued and increasing use of traditional stoves throughout the study, the decline in reported intervention stove use, and the lack of effect on personal exposure to PM_2.5_ among both household cooks and children. Our results are in contrast with 2 recent biomass-burning stove intervention studies in Senegal and Peru, which found no effect on self-reported respiratory symptoms among cooks and/or children under 5 [47,48]. Previous studies have suggested that near-exclusive use is required for health effects [49], although the emissions profile of the stove and where it is used are also critically important. A recent trial in Malawi examined a higher-tier, cleaner-burning forced draft cookstove than the rocket natural draft stove in our study and found no impact on child pneumonia [15]. However, the outcome was only diagnosed at health facilities, and impact on less severe infections among children not seeking facility care was not reported.

There was also a reduction in reported burns among children. This protective effect associated with improved stove dissemination was also observed in recent trials in Malawi and Nepal [15,50]. In sub-Saharan Africa, burns most often occur at home, affect young children, and are caused by hot liquids and open flames that are often present during cooking [51]. The intervention stove concentrates and shelters open flames more than traditional three-stone fires and may explain the reduced occurrence of burns observed in this study.

Moving some cooking activities outdoors, a key behavior change message delivered and reinforced by the implementer (DelAgua Health and Rwanda MOH), could have contributed to the reported respiratory benefits: just over half of households reported following the promoters’ instructions to use the intervention stove outdoors when possible, and more than a third of intervention households reported that their primary cooking location was outdoors. Previous studies have found associations between child ARI and cooking location [52] and cooking area ventilation [53]. Indeed, a multicountry study found that outdoor cooking in Africa during the dry season was protective against ALRI-related mortality [54]. In a previous trial in rural Rwanda, we found that the intervention stove resulted in a significant reduction in cooking area PM_2.5_, particularly if the intervention stove was used outdoors [27], and it is possible that moving cooking activities outdoors has greater benefit for young children that we were unable to measure. The intervention stove may have the most benefit when used outdoors in rural areas where there is low housing density and minimal smoke intrusion from neighbors and other sources; this will be investigated in further per-protocol analyses of data from the study cohort. Our study did not include source apportionment, which would help determine whether differences in PM_2.5_ composition were associated with the observed effects on ARI.

Another possible explanation for the effects on ARI in the absence of reductions in PM_2.5_ is a potential synergistic effect from the reduction in diarrhea. Other studies in low-income settings have found that recent diarrhea increases the risk of ALRI [55,56]. The potential synergistic effects of improved water quality on respiratory health warrants further investigation utilizing biomarkers and more objective indicators of exposure and infection.

This study has a number of limitations. Use of the intervention filter and stove was reported and only observed at the time of the enumerator’s visit and could be subject to responder and observer bias. Use of the intervention could have been over-reported, and consumption of unfiltered water might have been underreported. A sensor-based study among a subsample of our study households in the first 6 months after receipt of the intervention found that reported intervention use was higher than confirmed use [57]. On the day prior to retrieval of the sensor used in the substudy, among 86 households, 67.4% of households reported using the filter at least once, but filter use was only detected in 37.2% of households. Similarly, intervention stove use was also over-reported: among 89 households, 84.2% of households reported stove use, but the sensors only detected use in 37.1% of households [57]. Long-term monitoring of the intervention using sensors would provide a more objective indication of usage patterns and could also assist implementers as they seek to maximize exclusive intervention use and realize potential health and environmental benefits.

A limitation of this study—and many water, sanitation, and hygiene (WaSH) and stove evaluations—is the reliance on caregiver-reported episodes of diarrhea and/or ARI as an outcome in unblinded studies [58]. Evidence of reporting (courtesy and social desirability) bias is strongly suggested by the fact that pooled estimates from masked household water treatment trials show no protective effect [12]. Although the secondary outcome of IMCI-defined pneumonia and severe pneumonia incorporates more objective measurements, this too is susceptible to bias since fast breathing was only measured among children with reported cough, and the identification of chest indrawing, number of breaths, and danger signs may be subject to enumerator bias. We previously attempted unsuccessfully to blind participants by using a sham LifeStraw filter [59]. Like other studies, we have previously used anthropometry measures and identification of pathogens in stools as more objective measures of intervention effect. However, recent evidence of differences in intervention effects on diarrhea compared to stunting have raised questions about the extent to which these are linked [42,60], and the assessment of enteric pathogens in stools is costly and not always predictive of diarrhea [44,61].

In this study, we believe our estimates of the impact of the intervention on health are corroborated by 4 factors. First, our results for reported diarrhea are consistent with the effect we previously reported on reduced seroprevalence of IgG response against *Cryptosporidium* among children under 2 years of age [46]. Second, a recent analysis of multiple field studies showed a dose–response relationship between water quality and reported diarrhea, providing some evidence of the validity of caregiver-reported diarrhea [62]. Third, episodes of caregiver-reported diarrhea and ARI were most common in children under 2 years of age and generally declined with increasing age, as would be expected (S2 Table) [7,63]. Fourth, there was a greater impact on reported visits to health facilities for diarrhea compared to any reported diarrhea in the last 7 days (regardless of care-seeking), and facility visits are arguably a more objective and severe outcome. Furthermore, the null effect we observed for reported care-seeking from health facilities for ARI, compared with the impact on care-seeking for diarrhea (and ARI treated by CHWs), might indicate greater impact on mild versus more severe ARI and may support the accuracy of the self-reported outcomes. Our primary definition of ARI was broadly defined a priori as cough accompanied by rapid or difficulty breathing, although a more restrictive definition using DHS-defined criteria to limit cases to only those with reported chest involvement was examined. Even IMCI-defined pneumonia and severe pneumonia have an emphasis on sensitivity rather than specificity, and it is difficult to diagnose clinically significant pneumonia in resource-challenged settings. Our study design calls for securing additional data directly from CHWs and clinical records [23]. This should further assist in assessment of the impact of the intervention on diarrhea and respiratory infections, especially the more serious cases that cause patients to present at clinics.

The results from this study should have important policy implications in Rwanda and beyond. They are in contrast to another recent evaluation of a community health club program in the country to promote improved WaSH and nutrition, in which no effect was observed on measures of drinking water quality, reported diarrhea, or stunting [64]. However, drinking water is a major source of fecal exposure in Rwanda [22], as it is in many low-income countries [3], and our study findings are consistent with those of other studies and meta-analyses that show similar risk reductions with household water treatment, particularly methods involving point-of-use filtration [12]. To address the limitations and challenges of boiling water—which is currently recommended within Rwanda’s national Community-Based Environmental Health Promotion Program (CBEHPP)—current research with the support of the Rwanda MOH is exploring the feasibility of promoting filters to vulnerable households through the CBEHPP program, and these results will inform future national initiatives.

While we have focused on diarrhea, ARI, and other health symptoms among children <5 years of age, other plausible benefits from the exclusive adoption and correct use of water filters and cleaner-burning cookstoves could be explored through further study, such as time-saving among women and children, who are most often tasked with water and fuel collection and cooking activities. Over 98% of people in Rwanda still rely on solid biomass fuel for cooking, and the availability and uptake of cleaner fuels needed for optimal health impacts in the near future is likely to remain out of reach for many, especially among the rural poor, unless access is accelerated. Household water treatment—using technologies that are effective against the full range of waterborne pathogens, provide longer-term protection without the need to replace consumables, and are acceptable to and actually used by vulnerable populations on a sustainable basis—offers a solution that should be promoted on an interim basis until these populations can benefit from solutions that many of us take for granted, such as safe, reliable, and adequate supplies of piped water to the home.

## Supporting information

S1 Table Exposure subsample characteristics at baseline. (DOCX)

S2 Table Prevalence of reported 7-day diarrhea and 7-day ARI by age group (in months) and treatment arm during follow-up. ARI, acute respiratory infection.
(DOCX)

S1 Checklist CONSORT checklist. (DOCX)

## Abbreviations

ALRI: acute lower respiratory infection
ARI: acute respiratory infection
CBEHPP: Community Based Environmental Health Promotion Program
CHW: community health worker
CI: confidence interval
DHS: Demographic and Health Survey
GEE: generalized estimating equation
HAP: household air pollution
H-PEM: Harvard Personal Exposure Monitor
ICC: intracluster correlation
ICCM: Integrated Community Case Management
IgG: immunoglobulin G
IMCI: Integrated Management of Childhood Illness
MOH: Ministry of Health
MUAC: middle-upper arm circumference
PM_2.5_: fine particulate matter less than 2.5 micrometers in diameter
PPES: probability proportional to estimated size
PR: prevalence ratio
SODIS: solar disinfection
TTC: thermotolerant coliform
WaSH: water, sanitation, and hygiene
WHO: World Health Organization
