# Supplementary material for: Effects of a large-scale distribution of water filters and natural draft rocket-style cookstoves on diarrhea and acute respiratory infection: A cluster-randomized controlled trial in Western Province, Rwanda
Source: PLoS Med. 2019 Jun 3;16(6):e1002812. doi: 10.1371/journal.pmed.1002812 (PMC6546207; doi:10.1371/journal.pmed.1002812)
Supplement: S1 Table — (DOCX) [file pmed.1002812.s001.docx]

**Supplemental Table 1.** Exposure sub-sample characteristics at baseline.

|  | **Intervention (%)** | **Control (%)** |
| --- | --- | --- |
|  | **N=112 houses** | **N=112 houses** |
| **Participant characteristics** |  |  |
| Age of primary cook – years (SD) | 34.4 (10.5) | 31.1 (9.2) |
| Gender – female | 112 (100) | 112 (100) |
| Attended school | 81/112 (72.3) | 70/112 (62.5) |
| Age of child – months (SD) | 34.2 (8.5) | 36.0 (7.4) |
| Gender | 54 (48.2) | 58 (51.8) |
| **Household characteristics** |  |  |
| Mean # of household residents (SD) | 5.2 (1.8) | 5.2 (1.8) |
| Owns house | 104/112 (92.9) | 102/112 (91.1) |
| Has electricity | 6/112 (5.4) | 4/112 (3.6) |
| Owns mobile phone | 52/112 (46.4) | 41/112 (36.6) |
| Owns livestock | 59/111 (53.2) | 66/112 (58.9) |
| Floor material: Earth/sand | 105/112 (93.8) | 109/112 (97.4) |
| Mean number of rooms (SD) | 4.0 (1.4) | 3.9 (1.3) |
| **Cooking, lighting, and heating** |  |  |
| Current primary fuel: wood | 69/112 (61.6) | 56/112 (50.0) |
| Current primary fuel: straw/shrubs/grass | 43/112 (38.4) | 51/112 (45.5) |
| Current primary fuel: charcoal | 0/112 (0.0) | 4/112 (3.6) |
| Cooks 1 meal per day (%) | 30/112 (26.8) | 29/112 (25.9) |
| Cooks 2 meals per day (%) | 71/112 (63.4) | 77/112 (68.8) |
| Primary cooking location: Inside house – kitchen room | 35/112 (31.3) | 38/112 (33.9) |
| Primary cooking location: Inside house – other room | 19/112 (17.0) | 26/112 (23.2) |
| Primary cooking location: Outside | 6/112 (5.4) | 7/112 (6.3) |
| Primary cooking location: Inside separate kitchen | 52/112 (46.4) | 41/112 (36.6) |
| Has traditional three-stone fire and uses everyday | 93/107 (86.9) | 83/108 (76.9) |
| Has built-in wood-burning stove (without chimney) and uses everyday | 14/107 (13.1) | 22/108 (20.4) |
| Has traditional charcoal stove and uses every day | 0/107 (0.0) | 3/108 (2.8) |
| Use kerosene for lighting every day | 9/112 (8.0) | 4/112 (3.6) |
| Heated home in last 7 days | 13/112 (11.6) | 14/112 (12.5) |
|  |  |  |
| **48-hour personal exposure to PM_2.5_ - Cooks** | **N=81** | **N=86** |
| PM_2.5_ (μg/m^3^) mean (SD) | 329 (394) | 258 (218) |
| PM_2.5_ (μg/m^3^) median, IQR | 201, 121-357 | 202, 122-333 |
|  |  |  |
| **48-hour personal exposure to PM_2.5_ - Children** | **N=56** | **N=60** |
| PM_2.5_ (μg/m^3^) mean (SD) | 322 (286) | 224 (182) |
| PM_2.5_ (μg/m^3^) median, IQR | 229, 131-382 | 188, 114-290 |
