# Supplementary material for: Effects of a large-scale distribution of water filters and natural draft rocket-style cookstoves on diarrhea and acute respiratory infection: A cluster-randomized controlled trial in Western Province, Rwanda
Source: PLoS Med. 2019 Jun 3;16(6):e1002812. doi: 10.1371/journal.pmed.1002812 (PMC6546207; doi:10.1371/journal.pmed.1002812)
Supplement: S2 Table — ARI, acute respiratory infection. (DOCX) [file pmed.1002812.s002.docx]

**Supplemental Table 2.** Prevalence of reported 7-day diarrhea and 7-day ARI by age group (in months) and treatment arm during follow-up.

|  | **0-5mo^1^.** | **6-11mo.** | **12-23mo.** | **24-35mo.** | **36-47mo.** | **48-59mo.** | **All ages (0-59mo.)** |
| --- | --- | --- | --- | --- | --- | --- | --- |
|  | **N (%)** | **N (%)** | **N (%)** | **N (%)** | **N (%)** | **N (%)** | **N (%)** |
| 7-day diarrhea |  |  |  |  |  |  |  |
| Control | 15 (8.1) | 44 (18.7) | 144 (21.6) | 86 (13.6) | 65 (9.0) | 43 (6.7) | 397 (12.9) |
| Intervention | 10 (6.7) | 34(16.5) | 95 (16.2) | 53 (8.7) | 37 (5.3) | 20 (3.3) | 249 (8.7) |
| 7-day ARI |  |  |  |  |  |  |  |
| Control | 38 (20.2) | 53 (22.6) | 117 (17.6) | 90 (14.3) | 81 (11.3) | 62 (9.6) | 441 (14.3) |
| Intervention | 19 (12.7) | 28 (13.6) | 81 (13.8) | 47 (7.7) | 66 (9.4) | 42 (7.0) | 283 (9.9) |
